# Chemosensitivity to doxorubicin in primary cells derived from tumor of FVB/N-Trp53^tm1Hw1^ with TALEN-mediated Trp53 mutant gene

**DOI:** 10.1186/s42826-023-00175-2

**Published:** 2023-10-20

**Authors:** Woobin Yun, Ji Eun Kim, You Jeong Jin, Yu Jeong Roh, Hee Jin Song, Ayun Seol, Tae Ryeol Kim, Kyeong Seon Min, Eun Seo Park, Gi Ho Park, Hyun Gu Kang, Yeon Shik Choi, Dae Youn Hwang

**Affiliations:** 1 Department of Biomaterials Science (BK21 FOUR Program)/Life and Industry Convergence Research Institute, College of Natural Resources & Life Science, Pusan National University, Miryang, 50463 Korea; 2 Department of Laboratory Medicine, Yonsei University College of Medicine, Seoul, 03722 Korea; 3 Department of Veterinary Theriogenology, College of Veterinary Medicine, Chungbuk National University, Cheongju, 28644 Korea; 4 Department of Biomedical Analysis, Bio Campus of Korea Polytechnic, Nonsan, 32943 Korea

**Keywords:** Chemosensitivity, Doxorubicin, Trp53, Solid tumor, Ascetic tumor, G2 arrest

## Abstract

**Background:**

To evaluate the chemosensitivity to doxorubicin (DOX) in two primary cells derived from a tumor of FVB/N-Trp53^tm1Hw1^ knockout (KO) mice with TALEN-mediated Trp53 mutant gene, we evaluated the cell survivability, cell cycle distribution, apoptotic cell numbers and apoptotic protein expression in solid tumor cells and ascetic tumor cells treated with DOX.

**Results:**

The primary tumor cells showed a significant (*P* < 0.05) defect for UV-induced upregulation of the Trp53 protein, and consisted of different ratios of leukocytes, fibroblasts, epithelial cells and mesenchymal cells. The IC_50_ level to DOX was lower in both primary cells (IC_50_ = 0.12 μM and 0.20 μM) as compared to the CT26 cells (IC_50_ = 0.32 μM), although the solid tumor was more sensitive. Also, the number of cells arrested at the G0/G1 stage was significantly decreased (24.7–23.1% in primary tumor cells treated with DOX, *P* < 0.05) while arrest at the G2 stage was enhanced to 296.8–254.3% in DOX-treated primary tumor cells compared with DOX-treated CT26 cells. Furthermore, apoptotic cells of early and late stage were greatly increased in the two primary cell-lines treated with DOX when compared to same conditions for CT26 cells. However, the Bax/Bcl-2 expression level was maintained constant in the primary tumor and CT26 cells.

**Conclusions:**

To the best of our knowledge, these results are the first to successfully detect an alteration in chemosensitivity to DOX in solid tumor cells and ascetic tumor cells derived from tumor of FVB/N-Trp53^tm1Hw1^ mice TALEN-mediated Trp53 mutant gene.

## Background

An anthracycline drug, DOX is used in treating a wide spectrum of solid epithelial and mesenchymal tumors and acute leukemias [1]. During chemotherapy, the cytotoxic effects of the drug are mediated by various cellular mechanisms. Most of the mechanisms induce the inhibition of DNA topoisomerase II, which causes DNA strand breaks [1–3]. In some cases, DOX is known to stimulate the release of free radicals, as demonstrated in the isolated rat heart, although this production is prevented by the addition of superoxide dismutase (SOD) and catalase (CAT) [4]. Furthermore, DOX directly intercalates into the cellular DNA, disrupting the DNA structure and inhibiting the progress of topoisomerase II, thereby inducing the accumulation of DNA strand breaks [5]. Therefore, this intercalation significantly affects gene transcription and replication of DNA [6].

Many Dox-resistant tumor cells have developed, having numerous possible mechanisms including alteration of the glutathione level, reduction of cytochrome P450 (CYP450)-reductase, overexpression of multidrug resistance protein 1 (MDR1), and alteration of toperisomerase II activity [7–10]. Especially, the cytotoxicity of DOX is affected by the cellular status of Trp53 protein in several cells [6]. Resistance to DOX was significantly enhanced in fibroblasts derived from KO mice with targeted Trp53 gene, MCF-7/Adr breast cancer cells with some deletion within exon 5 of the Trp53 gene, KATOIII stomach cell line, and RCM-3 rectum cell line with some deletions in the Trp53 gene [6, 11, 12]. However, contradictory results have been observed in cells having a defective Trp53 gene, such as hematoma cells and glioma cells. DOX resistance was lower in Hep3B cells (IC_50_ = 7.1 μg/mL) with deleted Trp53 gene than in HepG2 cells (IC_50_ = 16.2 μg/mL) with wild type Trp53 gene [13]. Furthermore, no significant alterations in DOX resistance were observed in human LN-308 glioma cells with deleted Trp53 gene [14, 15]. Taking these results into account, recent studies have focused on the chemosensitivity and apoptotic response to DOX in various tumor cells with different Trp53 gene deletions.

In this study, we evaluated the chemosensitivity to DOX in solid and ascetic primary cells derived from a tumor of FVB/N-Trp53^tm1Hw1^ KO mice with TALEN-mediated Trp53 mutant gene. Our research approach, focusing on one of the deletions in Trp53, will provide insights into how it responds to DOX drug treatment. Particularly, this approach can provide scientifical evidences about TALEN-mediated deletion of Trp53 gene in DOX resistance. This is expected to contribute to both Trp53 mechanistic studies and cancer research on DOX drugs.

## Results

### Characterization of primary tumor cells derived from tumor of FVB/N-Trp53^tm1Hw1^ mice

**Fig. 1:**
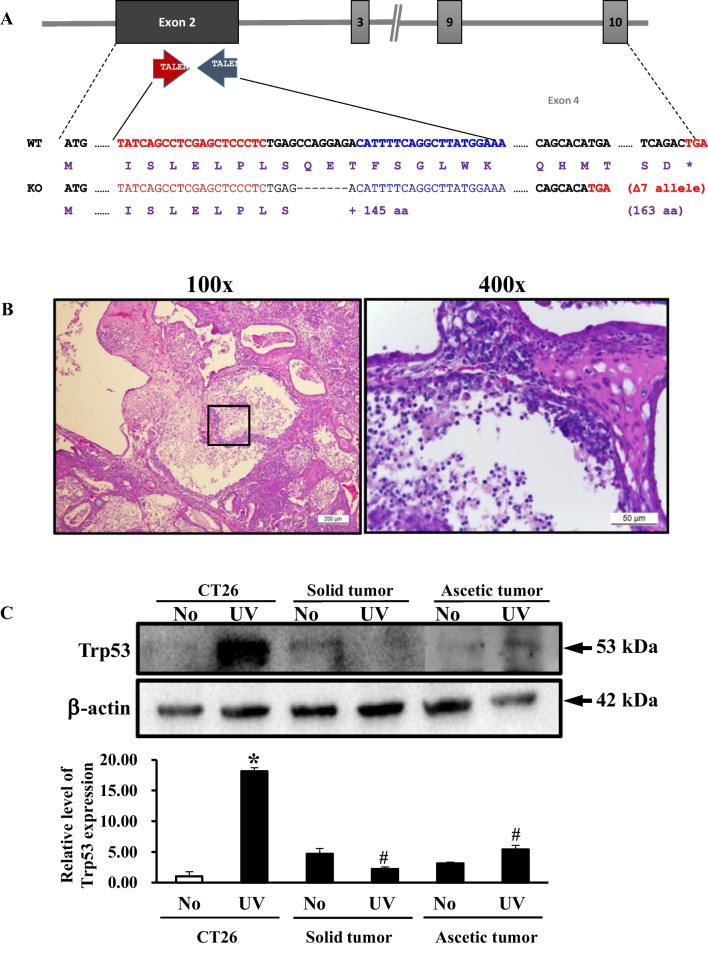
Design for Trp53 gene deletion, histopathology of solid tumor and Trp53 expression. **A** FVB/N-Trp53^tm1Hw1^ mice used in this study have the highly active TALENs specific to exon 2 of Trp53. **B** H&E stained sections of solid tumor from the FVB/N-Trp53^tm1Hw1^ mice were observed at 100 × (left column) and 400 × (right column) using a light microscope. Rectangles in the left column were magnified into the right column. **C** After UV radiation with 40 J, the cell homogenates were prepared from the primary tumor cells of the subset group, and transferred onto nitrocellulose membranes. The levels of Trp53 protein and β-actin were detected with specific antibodies, followed by horseradish peroxidase-conjugated goat anti-rabbit IgG. The intensity of each band was determined using an imaging densitometer. The data presented represents the means ± SD (n = 3). *, *p* < 0.05 relative to the No treated group. #, *p* < 0.05 relative to the CT26 cells

We first analyzed for the Trp53 expression and subpopulation of the two primary tumor cells to characterize their properties. The solid tumor and ascetic tumor were collected from the back and abdominal cavity of FVB/N-Trp53^tm1Hw1^ KO mice, respectively, and the solid tumor was determined to be an adenocarcinoma (Fig. 1B).

We observed that the expression of Trp53 protein was not upregulated in the primary tumor cells with TALEN-mediated mutant Trp53 gene after UV radiation, while they were upregulated in the CT26 cells with wild type Trp53 gene under same conditions (Fig. 1C). These results suggest that the primary tumor cells derived from FVB/N-Trp53^tm1Hw1^ mice are defective in the expressional regulation of Trp53 protein.

**Fig. 2:**
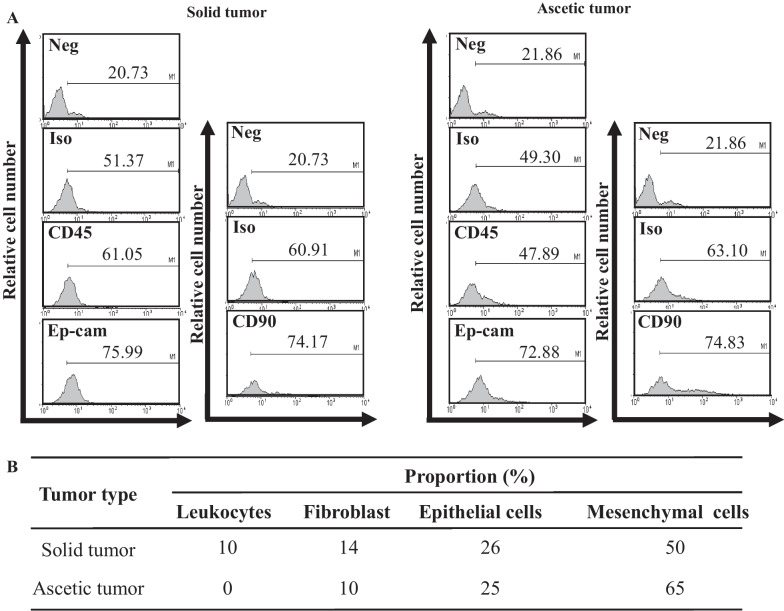
Composition analysis of the primary tumor cells derived from the FVB/N-Trp53^tm1Hw1^ mice. **A** After the collection of primary cells from solid tumor and ascetic tumor, the levels of several surface markers were determined by flow cytometric analysis using specific antibody such as CD45, Ep-cam and CD90. **B** Proportion of cells, including leukocytes, fibroblast, epithelial cells and mesenchymal cells, were calculated based on the data of flow cytometric analysis

We further analyzed the subpopulation of the primary cells derived from two different tumors of FVB/N-Trp53^tm1Hw1^ KO mice. To achieve this, the expression of marker proteins indicating leukocytes, epithelial cell, fibroblasts and mesenchymal cells were evaluated using FACS analysis. As showed in Fig. 2, the primary cells from the solid tumor was composed of 10% leukocytes, 26% epithelial cell, 14% fibroblasts and 50% mesenchymal cells, while the ascetic tumor was composed of 25% epithelial cell, 10% fibroblasts and 65% mesenchymal cells. These results indicate that the solid tumor and ascetic tumor derived from FVB/N-Trp53^tm1Hw1^ mice may be composed of a subpopulation of different cells, although three cell types were common.

## IC_50_ of primary tumor cells with TALEN-mediated mutant Trp53 gene

**Fig. 3:**
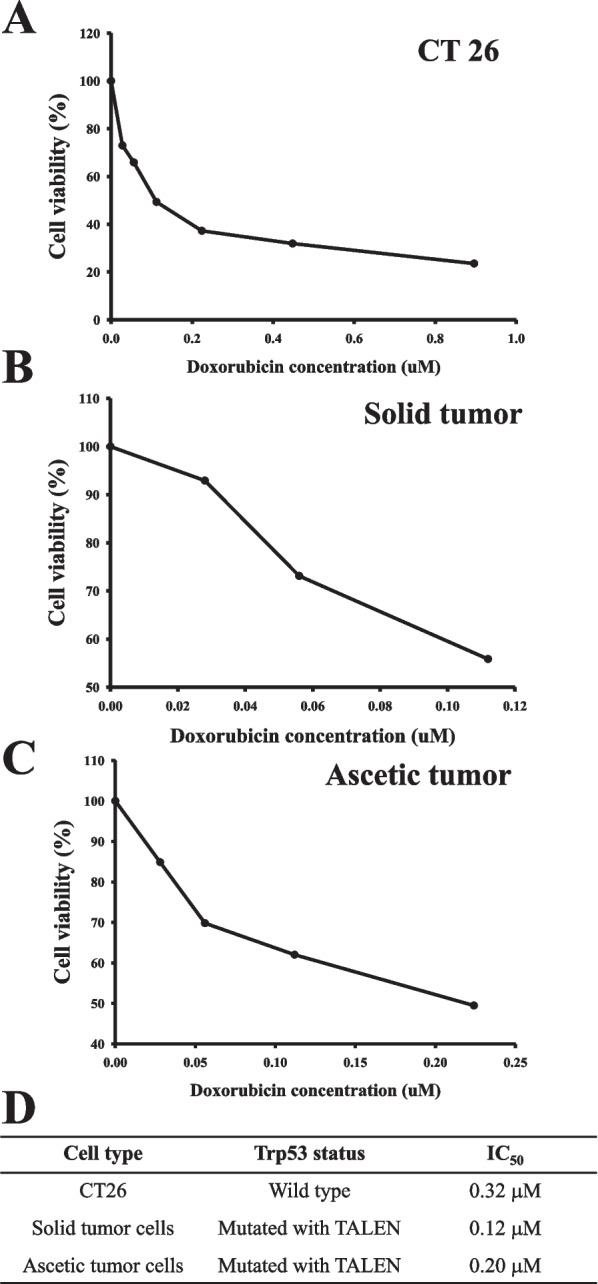
Cell viability of primary tumor cells to DOX. **A**-**C** After incubation of CT26, solid tumor cells and ascetic tumor cells with different concentrations of DOX for 24 h, the cell viability was determined by the MTT assay in triplicate. **D** The inhibitory concentration 50% (IC_50_) value is defined as the concentration of DOX that results in a 50% loss in cell viability

To determine the cytotoxic effects of DOX treatment on the cells with TALEN-mediated mutant Trp53 gene, IC_50_ levels were measured in CT26 cells, solid tumor cells and ascetic tumor cells exposed to various concentration of DOX. As shown in Fig. 3, the cell viability gradually decreased with increasing concentrations of DOX, although the decrease rate and response dose of DOX were varied. The IC_50_ values of CT26 cells, solid tumor cells and ascetic tumor cells were 0.32 μM, 0.12 μM and 0.20 μM, respectively. CT26 cells were the most DOX-resistant compared to the tumor cells derived from FVB/N-Trp53^tm1Hw1^ mice. Notably, the solid tumor cells and ascetic tumor cells were more sensitive to DOX (62.5% and 37.5%) than the CT26 cells (Fig. 3D). Therefore, these results suggest that the primary tumor cells with TALEN-mediated mutant Trp53 gene are more sensitive to DOX than CT26 cells with wild type Trp53 gene.

## Cell cycle arrest of primary tumor cells with TALEN-mediated mutant Trp53 gene

The Trp53 protein plays an important role at the G1 and G2 checkpoints during treatment with DNA-damaging agents such as UV, hydrogen peroxide and actinomycin [16]. To examine an alteration in the cell cycle arrest of primary tumor cells with TALEN-mediated mutant Trp53 gene after DOX treatment, the percentage of cells at various stages of the cell cycle were analyzed by flow cytometry, in all subset groups at 2, 4, 6, 12 and 24 h after exposure to IC_50_ of DOX.

**Fig. 4:**
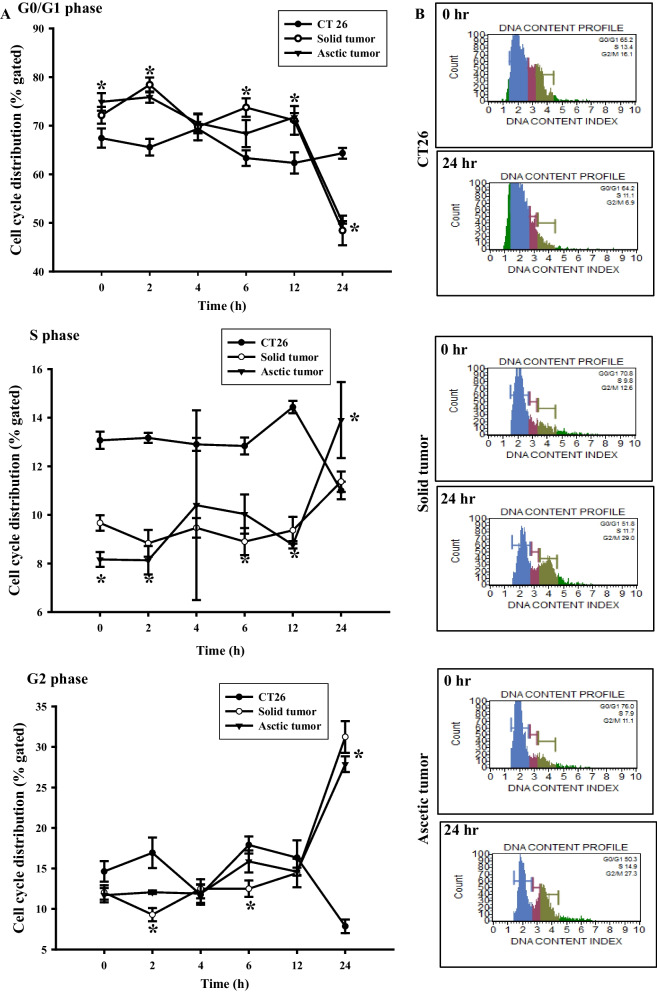
Cell cycle analysis after DOX treatment. The cell cycle was analyzed by flow cytometric analysis of the DNA content of nuclei of cells following staining with propidium iodide. After treatment with DOX, the number of cells in the G0/G1, S and G2/M stage was determined at each time point **A**. Data of FACS analysis of CT26 cells, solid tumor cells and ascetic tumor cells at 0 and 24 h **B**. The data presented represents the means ± SD (n = 3). *, *p* < 0.05 relative to the CT26 cells

We observed that the two primary tumor cells with TALEN-mediated mutant Trp53 gene showed different responses to DOX treatment compared to CT26 cell with wild Trp53 gene. Especially, the cell cycle of both primary cells was dramatically changed in the G0/G1 and G2 phases at 24 h, although there was no significant change from 0 to 12 h. A significant G2 arrest was observed in solid and ascetic primary cells treated with DOX for 24 h compared with CT26 cells (*P* < 0.05), while the number of G0/G1 cells was significantly decreased under same conditions (*P* < 0.05). However, the S phase showed no significant difference between primary tumor cells and CT26 cells at 24 h after DOX treatment (Fig. 4). These results therefore indicate that DOX treatment induces G2 arrest in the solid and ascetic primary cells with TALEN-mediated mutant Trp53 gene.

## Apoptotic response of primary tumor cells with TALEN-mediated mutant Trp53 gene

**Fig. 5:**
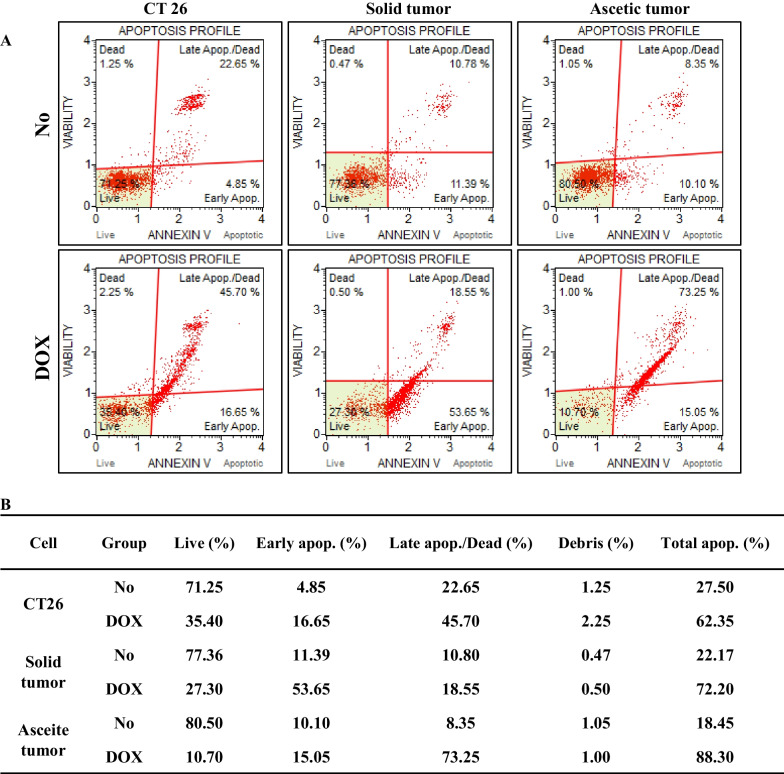
Number of Annexin V stained cells after IC_50_ DOX treatment. Fluorescence Activated Cell Sorter (FACS) data represents four independent phases (live, dead, early apoptosis, and late apoptosis). The data presented represents the means ± SD (n = 3)

**Fig. 6:**
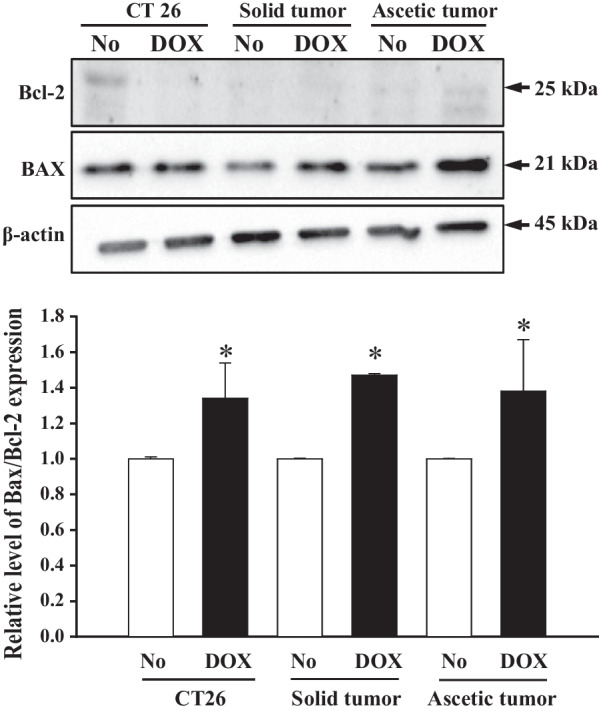
Expression of Bax and Bcl-2 protein after DOX treatment. After transfer of the cell homogenates onto nitrocellulose membranes, the expression levels of Bax, Bcl-2 and β-actin were detected with specific antibodies, followed by horseradish peroxidase-conjugated goat anti-rabbit IgG. The intensity of each band was determined using an imaging densitometer. The data presented represents the means ± SD (n = 3). *, *p* < 0.05 relative to the No treated group

Finally, to investigate the apoptotic response of primary tumor cells with TALEN-mediated mutant Trp53 gene after DOX treatment, the number of apoptotic cells, and the expression levels of Bax/Bcl-2 were measured in DOX treated CT26 cells, solid and ascetic primary tumor cells. As shown in Fig. 5, total apoptotic cells after DOX treatment were higher in the two primary tumor cells as compared to CT26 cells. The number of early apoptotic cells was especially increased in the DOX-treated solid primary tumor cells, while the number of late apoptotic cells was increased in DOX-treated ascetic primary tumor cells. However, Bax/Bcl-2 expression was maintained at a constant level in all groups, although their level was higher in the DOX-treated group than No-treated group (Fig. 6). These results suggest that DOX treatment enhances the apoptotic response in the solid and ascetic primary cells with TALEN-mediated mutant Trp53 gene, regardless of the expression levels of related proteins.

## Discussion

The resistance of tumor cells against DOX is a major problem encountered during cancer therapy. This process includes various regulators such as glutathione, CYP450 reductase, NADPH, MDR1 protein and toperisomerase II [7–9]. Especially, Trp53 was suggested as a possible linker for the development of DOX resistance, since the increased expression of apoptotic Fas-R after DOX treatment is regulated by the Trp53 protein [17–19]. Several studies have therefore investigated the chemosenstivity to DOX in various cells with Trp53 mutation and deletion [6, 13]. The present study provides novel results about the chemosensitivity and apoptotic response against DOX treatment in primary cells with TALEN-mediated mutant Trp53 gene. Particularly, primary cells from a solid tumor and ascetic tumor showed low levels of IC_50_ to DOX, increased induction of S and G2 arrest, decline of DNA fragmentation, and upregulation of apoptotic proteins after DOX treatment. Thus, our results provide additional evidences about the role of TALEN-mediated mutant Trp53 gene in DOX resistance during cancer therapy.

The IC_50_ value is defined as the DOX concentration required to decrease cell survival by 50% [11]. An alteration in this value is closely associated with mutation of the Trp53 gene and cell types. The IC_50_ levels are decreased in HepG3 cells (7.1 ug/mL) with deleted Trp53 gene, compared with HepG2 (16.2 ug/mL) cells having wild type Trp53 gene [13]. However, the levels were higher in mouse fibroblast deficient in Trp53 [6] as well as in KATOIII and RCM3 gastrointestinal cancer cell lines with Trp53 gene deletion, than in cells with wild type Trp53 gene [12]. In addition, a similar increase on IC_50_ value was observed in MCF-7/Adr human breast cancer cells with exon 5 deletion in the Trp53 gene [11]. In the current study, the IC_50_ value to DOX was measured in primary cells derived from two different tumors of FVB/N-Trp53^tm1^^Hw1^ mice with TALEN-medicated Trp53 mutant gene. The value in solid tumor cells and ascetic tumor cells decreased by 37.5% and 62.5% compared with CT26 cells with wild type Trp53 gene. These results are in complete agreement to a previous study, which showed decrease of the IC_50_ value (56.2%) in HepG3 cells with deleted Trp53 gene [13]; however, three other studies have shown different results [6, 11, 12]. We thought that these differences are attributable to the responding cells type and deletion site of the Trp53 gene, although a comprehensive direct comparison test is required to compare all results at the same time.

Trp53 activation following DNA damage regulates the cell cycle arrest, repairs damaged cells and cell apoptosis through the transcriptional regulation of various proteins [20]. During the regulation of cell cycle, the G1/S checkpoint arrested prior to DNA replication is considered as being entirely Trp53 dependent, while the G2/M checkpoint arrest occurs before mitosis and can be regulated by multiple pathways, including Trp53 [21]. In the process of the G2/M checkpoint arrest, Trp53 repressed the expression of cdc25, cyclin B, p21, 14–3-3 sigma protein and GADD45 [22–25]. Also, significant arrest of G2/M after DOX treatment was observed in several cells with Trp53 gene deletion. HepG3 cells showed G2/M arrest soon after DXO treatment [13]. However, in KATOIII and RCM3 cells with Trp53 gene deletion, the G2 arrest increased by 20.5% at a low dose (5 µM) of DOX, while G0/G1 and S arrest were enhanced by 7% and 13.6% at higher doses (55 µM) of DOX [12]. In this study, the primary cells derived from solid tumor and ascetic tumor of FVB/N-Trp53^tm1Hw1^ mice with TALEN-medicated Trp53 mutant gene showed G2 and S arrest at 24 h after DOX treatment (at less than 5 µM), while the cell percentage of G0/G1 phase were decreased. These results are similar to previous studies wherein G2, G0/G1 and S arrest were enhanced in KATOIII and RCM3 cells treated with 5 µM and 50 µM DOX [12]. We are the first to report that tumor cells with TALEN-medicated Trp53 mutant gene successfully induce S and G2 arrest after treatment with IC_50_ concentrations of DOX.

The Trp53 protein controls apoptosis in response to abnormal proliferation signals and stress including DNA damage, through the regulation of various proteins such as Bax, Bcl-2 and Fas-R [6, 26]. DOX, a chemotherapeutic agent, induces sever stress causing enhancement of Trp53 protein levels, resulting in an increase of Fas-R expression on the surfaces of some cells [27]. In Trp53 deficient mouse fibroblast cells, the Fas-R expression was induced by DOX treatment although their level was lower than that of cells with wild type Trp53. However, no significant changes in the expression of Bcl-2 and Bax in Trp53 wild type and knockout cells were seen [6]. A similar result was observed in the present study. After DOX treatment, no significant changes were observed in the Bax and Bcl-2 protein expression levels in both Trp53 wild type and KO cells. These accumulation of similar pattern of Bcl-2 family members may support that DNA damage including DOX treatment on both Trp53 wild type and Trp53 KO cells cause Bcl-2 family member-independent apoptosis.

Utilizing TALEN-mediated Trp53 deletion mice, we discerned diverse molecular alterations in Trp53 upon exposure to DOX. Nevertheless, a comprehensive elucidation of the underlying causes for the conflicting outcomes in Trp53 responses to DOX remains unclear. Additionally, similar to previous research, it appears necessary to elucidate through further studies why there is no significant change in Bax and Bcl-2 protein expression in Trp53 knockout cells following DOX treatment when compared to Trp53 wild-type cells.

## Conclusions

Taken together, these results show, for the first time, that TALEN-mediated mutant Trp53 gene induces a significant alteration in the chemosensitivity to DOX in primary cells derived from solid and ascetic tumors of FVB/N-Trp53^tm1Hw1^ mice. Especially, we show that DOX treatment induces a decrease in the IC_50_, enhancement of G2 arrest, and increase of apoptotic cells in Trp53 deficient cells as compared to the Trp53 wild type cells. However, the present study provides limited information since the primary tumor cells derived from only KO mice were examined. Furthermore, trials for FVB/N-Trp53^tm1Hw1^ mice as novel cancer model are necessary to clarify the alteration on the chemosensitivity and apoptotic mechanism after DOX treatment.

## Methods

### Cell culture

CT26 cell which is the mice colon carcinoma containing wild type Trp53 gene was purchased from the Korean Cell Line Bank (Seoul, Korea) [28]. These cells were grown in monolayers in Dulbecco’s Modified Eagle’s Medium (DMEM, GIBCO, Grand Island, NY, USA) supplemented with 10% fetal bovine serum (FBS; Invitrogen, Carlsbad, CA, USA) and antibiotics (100 unit/mL penicillin and 100 μg/mL streptomycin; GIBCO). Also, two primary tumor cell lines, derived from a solid tumor and ascites fluid, were maintained in DMEM culture media containing 10% serum and 1% penicillin and streptomycin. All cultures were incubated at 37°C in a humidified incubator containing 5% CO_2_ in air.

## Harvest of primary tumor cells

FVB/N-Trp53^tm1Hw1^ mice used for harvesting primary tumor cells were kindly provided by the Department of Laboratory Animal Resources (Laboratory Animals Resources Bank) at the National Institute of Food and Drug Safety Evaluation (NIFDS, Chungju, Korea). These mice have 7 base pair deletions (55 to 61 nucleotides) and develop several types of tumors including lymphomas, teratomas and hemangiosarcomas, in various organs and abdominal cavity (Fig. 1A). Briefly, the solid tumor tissue (about 1–2 cm^3^) was collected from the back region of FVB/N-Trp53^tm1Hw1^ mice; all non-tumor tissue and necrotic tumor tissue was separated from the tumor using sterile sharp forceps and scissor. The tumor was washed with 1× PBS and chopped finely, and harvested by centrifuging at 1,300 rpm for 5 min at room temperature. The pelleted material was re-suspended in 10 mL of Tumor Cell Digestion Solution (40 mg collagenase type 2, 40 mg trypsin inhibitor, 80 mg BSA in 40 mL of DMEM medium), and incubated at 37°C for 40 min with 140 rpm agitation. This suspension was passed through a 70 μm mesh cell strainer, and the primary tumor cells were harvested by centrifugation at 1000 rpm for 10 min. After discarding the supernatant, the resulting cell pellet was resuspended in DMEM culture media containing 10% serum and 1% penicillin and streptomycin.

To collect the ascetic tumor cells from the abdominal cavity, ascites were first recovered from FVB/N-Trp53^tm1Hw1^ KO mice using a syringe. The primary tumor cells were then harvested by the centrifugation at 1,000 rpm for 10 min.

## Flow cytometry analysis

The subpopulation of tumor cell derived from the two tumors of FVB/N-Trp53^tm1Hw1^ mice were analyzed using FACS. Briefly, CT26 cells, solid tumor cells and ascetic tumor cells were seeded at a density of 1x10^6^ cells/10 mL in 100 mm dish, and cultured for 24 h in a 37°C humidified incubator. The harvested cells were then divided into five subgroups: negative control (Neg), isotype against CD45 and Ep-CAM, CD45 group for leukocytes, Ep-CAM for epithelial cells, isotype against CD90 and CD90 group for fibroblasts. The cells of each group were treated with their specific primary antibodies: PE Rat IgG2b, ĸ Isotype ctrl (BioLegend), PE anti-mouse CD45 antibody (BioLegend, San Diego, CA, USA), purified anti-mouse CD90 antibody (BioLegend), and PE anti-mouse Ep-CAM antibody (Ep-CAM, BioLegend), respectively, followed by incubation for 10 minutes at room temperature. After washing with 1x PBS, the cells for CD90 staining were treated with PE goat anti-rat Ig antibody (BD bioscience, San Jose, CA, USA), while other groups were processed without further treatment. Florescent intensity of each cell was analyzed using FACS (Milipore Co., Billerica, MA, USA). Furthermore, the number of mesenchemal cells were calculated by subtracting the combined number of leukocytes platelets, fibroblasts and epithelial cells from the total cells number.

## UV treatment

Briefly, CT26 cells, solid tumor cells and ascetic tumor cells were seeded at a density of 1 × 10^6^ cells/10 mL in each culture dish, and grown for 24 h in a 37°C humidified incubator. Each cell were divided into one of two groups: No treated group and UV treated group. The cells of UV treated group were treated with UV irradiation (40 J/dish) using an Omnicure S1500 (Poly Dispensing System, Orgeval, France), while No treated group did not received any treatment. After then, these cells were further incubated for 12 h in 37°C humidified incubator. Finally, the cells of each group were harvested with centrifugation and used to western blot analysis.

## Determination of IC_50_ using MTT assay

Cell viability was determined using the tetrazolium compound 3-[4,5-dimethylthiazol-2-yl]-2,5-diphenyltetrazolium bromide (MTT) (Sigma-Aldrich Co., MO, USA). Briefly, tumor cells were seeded at a density of 0.5 × 10^4^ cells/0.2 mL complete medium, and grown for 24 h in a 37°C incubator. On attaining 70–80% confluence, cells were treated with various concentrations of DOX (0.02, 0.04, 0.06, 0.08, 0.1, 0.12, 0.14, 0.16, 0.18, 0.2, 0.4, 0.6, 0.8 and 1 μM) for 72 h. Following incubation, the supernatants were discarded, after which 0.2 mL of fresh DMEM media and 50 μl of MTT solution (2 mg/mL in PBS) were added to each well. The cells were then incubated at 37°C for 4 h. The resulting formazan precipitate was dissolved in DMSO, and absorbance was read at 570 nm using a Molecular Devices VERSA max Plate reader (Sunnyvale, CA, USA). The morphological features of cells in each treated group were also observed microscopically (Leica Microsystems, Herbrugg, Switzerland). The IC_50_ value was determined from the dose response curves using multiple doses of DOX. This value was defined as the dose of DOX resulting in 50% loss of cell viability relative to untreated cells after DOX treatment.

## Cell cycle assay

Cell cycle analysis was done using a Muse™Cell Cycle Kit (MCH100106, Millipore Co., Billerica, MA, USA), according to the manufacturer’s instructions. Briefly, tumor cells in each group were divided into 6 subgroups (2.5 × 10^6^ cells/dish), following which they were cultured in DMEM medium containing 1% FBS in order to synchronize the cell cycles. After 12 h, cells were exposed to the respective IC_50_ of DOX (CT26 cells, 0.32 µM; solid tumor cells, 0.12 µM; ascetic cells, 0.20 µM) for variable durations (0, 2, 4, 6, 12 and 24 h). Total cells from the subgroups were harvested by centrifugation at 3,000 × g for 5 min, and fixed with 70% EtOH at -20°C for 3 h. After washing the fixed cells with 1× PBS, 200 μl of Cell Cycle Reagent was added to the suspension. Following incubation at 37°C in a CO_2_ incubator for 30 min, cell cycles were analyzed using FACS (Millipore Co., Billerica, MA, USA).

## Western blot

Total cell protein was extracted using the Pro-Prep Protein Extraction Solution (iNtRON Biotechnology, Seongnam, Korea) and quantified using a SMARTTM BCA Protein Assay Kit (Thermo Scientific, Waltham, MA, USA) for western blot. The proteins were separated by 4–20% sodium dodecyl sulfate-polyacrylamide gel electrophoresis (SDS-PAGE) for 2 h, after which the resolved proteins were transferred to nitrocellulose membranes for 2 h at 40 V. Each membrane was then incubated separately, overnight at 4°C, with the following primary antibodies: Bax antibody (Abcam, Cambridge, UK), Bcl-2 antibody (Thermo Fisher Scientific, Waltham, MA), Trp53 antibody (Abcam) and anti-actin antibody (Sigma-Aldrich Co.). The membranes were then washed with washing buffer (137 mM NaCl, 2.7 mM KCl, 10 mM Na_2_HPO_4_, and 0.05% Tween 20) and incubated with 1:1,000 diluted horseradish peroxidase (HRP)-conjugated goat anti-rabbit IgG (Invitrogen, Carlsbad, CA, USA) at room temperature for 1 h. Finally, all membrane blots were developed using Amersham ECL Select Western Blotting detection reagent (GE Healthcare, Little Chalfont, UK). The chemiluminescence signals that originated from specific bands were detected using FluorChemi^®^FC2 (Alpha Innotech Co., San Leandro, CA, USA).

## Statistical analysis

A t-test, a statistical test that is used to compare the means of two groups, was used to identify significant differences between No and UV treated groups, Neg group and marker antibody staining group, and CT26 cells and primary tumor cells. All values are reported as the mean ± standard deviation (SD), and a *p* value of < 0.05 was considered significant.

## Data Availability

All the data that support the findings of this study are available on request from the corresponding author.

## Abbreviations

DOX: Doxorubicin
KO: Knockout
TALEN: Transcription activator-like effector nuclease
UV: Ultraviolet radiation
IC_50_: The half maximal inhibitory concentration
DNA: Deoxyribonucleic acid
FACS: Fluorescence-activated cell sorter
DMEM: Dulbecco’s modified Eagle’s medium
FBS: Fetal bovine serum
CO_2_: Carbon dioxide
BSA: Bovine serum albumin
Neg: Negative control
MTT: Tetrazolium compound 3-[4,5-dimethylthiazol-2-yl]-2,5-diphenyltetrazolium bromide
DMSO: Dimethyl sulfoxide
BCA: Bicinchoninic acid
SDS-PAGE: Sodium dodecyl sulfate–polyacrylamide gel electrophoresis
NaCl: Sodium chloride
KCl: Potassium chloride
Na_2_HPO_4_: Sodium phosphate dibasic anhydrous
HRP: Horseradish peroxidase
